# Annotations

**Published:** 1895-08-17

**Authors:** 


### ANNOTATIONS.

### The Fountain Hospital.

The Local Government Board has sent a very strongly worded letter to the Asylums Board in regard to what they consider the excessive and uncontrolled expenditure upon the Fountain Hospital, pointing out that while the Tottenham Hospital was erected at a cost of £119 per bed the Fountain Hospital cost £288 per bed. An explanation of this discrepancy was asked for. The letter also stated that the Local Government Board were distinctly of opinion that, looking to the large expenditure of public funds which was involved, the Managers were not justified in leaving the matter in the hands of one man with such undefined directions as to his proceedings, and the less so when, as in the case of an architect, the remuneration was by a commission on the amount expended. In answer to this letter a long communication has been prepared by the General Purposes Committee, pointing out the difficulties which the Managers have met with in obtaining sites, the extremely pressing nature of the crisis in which the work was undertaken, the fact that much of the expense was due to the expedition with which the work had to be accomplished, and to the superior and more permanent nature of the Fountain buildings as compared with those erected at Tottenham. The Managers complain that the strictures of the Board will tend to weaken their hands in dealing with infectious disease, and deprive them of public confidence and support. That may be so, but no one can read the correspondence without being painfully conscious of the fact that such hurried construction as is here described, the building of hospitals against time, is a process necessarily involving wasteful extravagance, and regretting that two such important public bodies could not, in a matter of urgent public need, have so worked together as to render such extravagance unnecessary.

### Radcliffe Infirmary, Oxford.

The proceedings at the quarterly general Court of the Governors of this institution at the end of last month, though satisfactory in some respects, still leave much to desire. An imperfect attempt has been made to institute a personal canvas throughout the city of Oxford, and the results, under all the circumstances, are satisfactory. They prove that the people of Oxford are prepared to give annual subscriptions to the Infirmary, providing the managers show reasonable ability in the conduct of its affairs. At one time we were disposed to put down the sleepy condition into which the affairs of the Radcliffe Infirmary have fallen to the indifference of the citizens. Further experience of the proceedings at the quarterly court leads us however to the conclusion that the real fault lies with the Committee of Management. They evidently lack energy, administrative force, and courage. At the January court a special report was carried, in which the rearrangement of the duties of the staff and the appointment of a house governor responsible for all the details of administration was approved. We pointed out in The Hospital of May 11th that the Committee of Management appeared to have taken no steps in regard to this report, nor to have made any attempt to deal with the question of reorganisation, which pressed for settlement. The Oxford press joined us in the strong protest which we made in May, and it is remarkable that not a word was said as to this report or the reorganisation of the staff at the quarterly court in July. Are we to understand that the present committee are resolved to do nothing, preferring to leave the Radcliffe Infirmary in its present unsatisfactory condition? If so, we hope Lord Dillon will make a public statement to this effect, as in that case it may be necessary for the more intelligent of the governors to organise their forces in order to secure the appointment of a new committee of earnest and active men at the annual meeting. We believe it to be unparalleled for a committee to bring up a special report to the governors of a great hospital recommending the reorganisation of the staff, to carry that report at the governors' meeting, and then to do nothing in regard to its recommendations. In any case we do not think that those who have heretofore abstained from subscribing to the Radcliffe Infirmary will be justified in giving money to it under present circumstances, or possibly at all, until the report of the special committee has been acted upon, and the reorganisation of the staff has been carried out most completely and efficiently. Certainly the governors have been treated with contempt by the committee in regard to these changes, and it is contrary to the public interest that such conduct should be allowed to pass without protest which they can make effective by withholding their subscriptions until the committee show themselves to be worthy of confidence.

### Public Slaughter-houses and Sound Meat.

Sir Henry Littlejohn, the well-known Medical Officer of Health for the City of Edinburgh, made a statement at the annual congress of the British Institute of Public Health the other day, which may well excite surprise and envy in the minds of English sanitarians. He informed the congress that in Edinburgh private slaughter-houses had been abolished for 100 years. But what does this mean? It means at least two results of the highest sanitary importance. In the first place, all meat killed in public slaughter-houses can be competently inspected by the public authorities; and in the second place, the public slaughter-house, being under municipal control, need never become a nuisance and a danger from the neglect of thorough cleansing. Public slaughter-houses, in a word, mean sound meat for all classes of purchasers, and a wholesome and sanitary place for the killing and dressing of animal carcases for the market. What are the facts as regards many of the towns south of the Tweed? Here private slaughter-houses abound, with the two consequences that meat is seldom competently inspected, and that such slaughter-houses, from want of adequate cleansing, become centres of foul and pestilence-breeding smells. If we ask, "how can these things be" in an age in which science is everywhere active? the answer is that even science, like morals and religion, has to "speak with bated breath and whispering humbleness" in the presence of all powerful "vested interests." Now we are among those who have the sincerest respect for all sorts of vested interests which the law has allowed to grow up under its own shadow. Nevertheless, the safety of the State is the supreme law; and it is a disgrace to our civilisation and sanitary science that we have found no way of so dealing with vested interests as legitimately and honestly to abolish them in the higher interests of the public. Public slaughter-houses, with competent inspection of meat, ought to be the rule in every municipality in the civilised world. Every municipality which has failed to establish these must to that extent be considered a failure, unworthy of the science and civilisation of the nineteenth century.

